# Exploring auditory perception experiences in daily situations in autistic adults

**DOI:** 10.1177/13623613251391492

**Published:** 2025-11-14

**Authors:** Elena Sofia Silva, Linda Drijvers, James P Trujillo

**Affiliations:** 1 Radboud University Nijmegen, The Netherlands; 2 Max Planck Institute for Psycholinguistics, The Netherlands; 3 University of Amsterdam, The Netherlands

**Keywords:** auditory perception, autism, hearing, self-identifying autism, speech understanding

## Abstract

Autistic individuals often show differential sensory perception, including hypo- or hypersensitivities to sound. Previous research also suggests that autistic individuals often have difficulty processing intentional and affective cues in speech acoustics. However, general speech processing difficulties remain underexplored. We investigated self-reported auditory perception using the Speech, Spatial, and Qualities of Hearing Questionnaire among autistic (self-identifying (*n* = 18) and clinically diagnosed (*n* = 45)) and non-autistic adults (*N* = 66). The study was conducted in the Netherlands, but the questionnaire and call for participation were in English and open to anyone regardless of country of residence. Both clinically diagnosed and self-identifying individuals with autism reported significantly lower scores on the Speech, Spatial, and Qualities of Hearing Questionnaire score and on the Speech subscale compared with non-autistic individuals, indicating challenges in overall quality of auditory perception, speech comprehension. Clinically diagnosed individuals also showed lower scores on the quality and spatial subscales compared with non-autistic individuals. Post hoc analysis further suggested that speech hearing is particularly challenging for many autistic individuals. In addition, our finding that self-identifying and clinically diagnosed autistic individuals show similar patterns of hearing difficulties emphasizes the need for more inclusive research practices that collect the experiences of all the individuals in the autistic community in the study of sensory perception in autism.

**Lay abstract:**

Autistic individuals often have very different sensory experiences compared with non-autistic individuals. One anecdotally mentioned, but not well-researched phenomenon is difficulty processing what we are hearing. Rather than challenges related to language understanding, such as nonliteral or indirect language, autistic people may also have more difficulty making sense of the sounds of their environment. This may be hearing where particular sounds are coming from, or understanding what is being said, particularly in noisy situations. To bring more attention and clarity to this challenge, we asked autistic and non-autistic adults to fill out a short survey that measures one’s hearing experiences in daily life. We found that autistic individuals report more difficulty across several types of hearing, and most prominently regarding speech hearing, when compared with non-autistic individuals. This finding highlights that reports of auditory processing difficulties when there is no hearing loss are not niche experiences, but rather reflect a common experience in autistic adults. In addition, we found that clinically diagnosed and self-identifying individuals reported very similar experiences. This highlights the validity of self-identification/self-diagnosis for research aimed at understanding autistic experiences. This study, therefore, emphasizes the need for more research and awareness regarding auditory perception and hearing in autistic adults. The study also emphasizes the value of more inclusive research practices that collect the experiences of all individuals within the autism community.

## Introduction

Autistic^1^ individuals often face challenges associated with social interaction, as well as atypical sensory processing, including hyper- or hypo-reactivity to sensory input (American Psychiatric Association [APA], 2022). While the negative impact of sensory hyper- and hypo-reactivity on autistic individuals is known (Landon et al., 2016; Verhulst et al., 2022), it is less clear whether or in what way sensory processing differences contribute to challenges in ongoing social interaction. Particularly, the question of whether autistic individuals perceive difficulties relating to their auditory processing *abilities* rather than more generally to any hyper- or hypo-sensitivities has received little attention.

## Sensory perception in autism

Sensory challenges in autism are prevalent and multimodal, affecting individuals across age and ability levels (Leekam et al., 2006). These challenges can manifest as hypersensitivity or hyposensitivity to sensory inputs, such as sound, light, touch, taste, and smell. These differences are also strongly associated with self-reported stress in autistic adults (Harrold et al., 2024), as well as long-term mental health (MacLennan et al., 2022), and generally influence how autistic individuals engage with their environment (Poulsen et al., 2024). Beyond making particular situations or environments generally uncomfortable or intolerable, sensory sensitivities can also make it difficult to recognize and respond to socially relevant information embedded in voices and speech, ultimately reducing social reciprocity and making social interactions more challenging (Thye et al., 2018). According to Stiegler and Davis (2010), many individuals with autism experience distress, fear, and anxiety in response to certain frequencies or volumes of sound, and may have unpleasant physiological sensations as a result of autonomic and/or behavioral reactions to non-preferred sounds. However, at least some individuals can acquire the ability to successfully self-regulate their response (Stiegler & Davis, 2010). However, other autistic individuals may experience hyposensitivity, or a lower responsiveness to a particular sensory input than non-autistic individuals (Brinkert & Remington, 2020; Gonçalves & Monteiro, 2023). To date, little is known about how autistic individuals themselves perceive and navigate everyday hearing situations in comparison with non-autistic individuals. Understanding specific sensory symptoms, such as hyperreactivity to auditory stimuli or aversion to tactile experiences, can aid the development of improved targeted interventions for people on the autism spectrum (Pellicano, 2013).

One recent study using self-report measures has investigated clinically diagnosed autistic adults (primarily in the United Kingdom and Ireland) and found that broad autism traits are predictive of decreased sound tolerance as well as auditory perception difficulties (Bang & Igelström, 2024). Overall, the study found higher degrees of sound aversion in autistic compared with non-autistic individuals, and an association between communication-specific autism traits and multiple domains of auditory perception. Similarly, Yadon and Vonarx (2024) also found associations between autism traits and general sensory processing difficulties. While the study by Bang and Igelström provides an insightful analysis of how autistic traits relate to certain auditory experiences, an open question is whether different forms of auditory perception (e.g. speech hearing, spatial perception) are experienced differently by autistic adults. In addition, Bang and Igelström (2024) utilized full-length questionnaires. Assessing whether auditory perception challenges can be captured by short-form scales would be an insightful advance, as these provide less participatory burden on the autistic individuals, and could therefore be more effectively implemented in both research and clinical settings.

Furthermore, studies show different findings in the autistic population throughout the lifespan. For example, in comparison with age-matched controls, Kern et al. (2006) found that the loudness sensitivity of 104 autistic children and adults declined with age, eventually approaching control values. The authors suggested that this link represents the development of superior coping mechanisms and/or mature auditory processing areas to process sound intensity levels more effectively (Kern et al., 2006). Their findings indicated that sensory challenges associated with autism may improve with age, starting to improve approximately at 20 years old (Kern et al., 2006). Contrarily, Leekam et al. (2006) found variations in sensory difficulties among age groups in the general pattern of sensory response, and Chen et al. (2024) found that, in adults of 40–93 years old, sensory processing differences seem to increase with age (Chen et al., 2024). It is, therefore, important to assess or control for the role of age when determining how sensory challenges impact the daily lives of autistic individuals.

## Speech hearing, spatial hearing, and qualities of hearing

Auditory processing challenges may be particularly prominent in daily life, as it may impact the ability to tolerate busy social situations and comfortably speak with, and understand, others. Auditory processing differences may be categorized as challenges related to speech hearing, spatial hearing, and general quality of hearing. Regarding speech hearing, adults with autism may exhibit different activation patterns related to auditory cortical processing and difficulties with speech-in-noise recognition (Alcántara et al., 2004; Boddaert et al., 2003; Schelinski & Von Kriegstein, 2019). This suggests that the neural mechanisms underlying auditory processing may be different in autistic individuals, which may be contributing to difficulties in real-world listening situations. Critically, however, it is unclear to what extent autistic adults themselves experience challenges related to their hearing in daily situations. This problem can be seen as relating to *perceptual sensitivity* of autistic individuals, as described by the taxonomy of He et al. (2023).

Sturrock et al. (2022) conducted qualitative interviews in a small sample (*N* = 9) to explore autistic individuals’ experiences and challenges in speech hearing. In this study, most participants reported significant difficulties in perceiving speech when competing sounds were present. They highlighted that these difficulties were distinct from social challenges but could interact with them. Participants also identified compounding factors including a lack of understanding of the autistic person’s listening difficulties by their interaction partner (e.g. not realizing the impact of ambient noise on the autistic person’s ability to communicate). These additional factors had diverse and sometimes disabling impacts on aspects such as socializing, emotions, and career (Sturrock et al., 2022). The research by Sturrock et al. (2022) concluded that there was a need for further research to confirm, quantify, and understand the various forms of listening difficulty experienced by autistic individuals.

The ability of the auditory system to decipher and make use of the many spatial pathways that sound might take to reach the brain is known as spatial hearing. Through spatial hearing, the auditory system may locate a sound source and/or “unmask” sounds that would be muffled by background noise. In addition, it may direct attention toward or away from a sound source and, to some extent, define the characteristics of the listening environment (Culling & Akeroyd, 2010). While there is some evidence for autistic adults showing lower accuracy in a sound localization experiment than non-autistic adults (Visser et al., 2013), it is unclear to what extent such differences translate to real-life challenges experienced by autistic individuals.

Quality of hearing encompasses a broader area of study focused on how effectively individuals perceive various non-speech sounds. This domain includes listening effort, sound clarity, and the capacity to distinguish and identify sounds (Myhrum et al., 2023). The qualities of hearing extend to aspects such as music, pitch, and background sounds, providing insight into auditory experiences beyond speech. While autistic individuals’ auditory perception of pitch and music has been shown to be enhanced compared with neurotypical controls (Kellerman et al., 2005), it is unclear how autistic individuals experience the quality of their hearing in daily listening situations. This is an important facet of auditory processing and its relationship with social interaction, given that even if autistic individuals are able to understand speech in noisy environments and locate sounds in their environment, the quality of their hearing and the effort required to do so will certainly impact one’s ability and motivation to engage in social activities.

### Autism diagnosis

In order to gain a better understanding of how autistic individuals experience challenges related to auditory processing, it is important to critically consider who is being included in a given research sample. While a clinical diagnosis is considered the standard indicator of “being autistic” in the medical world and in much research, there are many individuals who self-identify either while waiting for formal diagnosis or without the intention of seeking formal diagnosis. This is because clinical diagnosis of autism in adulthood comes with many potential barriers, including costs, complexity of the healthcare system, or even difficulties or anxiety related to engaging with healthcare professionals regarding a diagnosis (de Broize et al., 2022; Fletcher-Watson, 2024; Lewis, 2016, 2017; Overton et al., 2023). Furthermore, while formal diagnosis is expected to open the possibility of services or support options, this is often not the case (Fletcher-Watson, 2024). Individuals who self-identify often find value in a better understanding of their own experiences, without the need for a formal diagnostic confirmation. It is, therefore, necessary to recognize the experiences of self-identifying individuals as valid and worthy of inclusion (see also Ardeleanu et al., 2024).

Considering the different experiences of those not officially diagnosed with autism and the highly heterogeneous nature of autism, it is also relevant to investigate whether there are any other differences in daily situations between self-identifying individuals and those with formal diagnoses. By incorporating self-identifying individuals into the sample, this study acknowledges their place within the autism research field, ensuring their contribution to our understanding of autism. This study compared these groups separately to determine whether any differences exist, with the hypothesis that there should be no differences in auditory perceptions between individuals clinically diagnosed with autism and those who self-identify. If this hypothesis is true, this would provide further support to continue to include both formally diagnosed and self-identifying autistic individuals in future research.

Finally, an additional aspect of the heterogeneous nature of autism is that co-occurring conditions such as attention deficit hyperactivity disorder (ADHD), anxiety, bipolar disorder, depression, Tourette’s syndrome, and others are present in nearly 75% of individuals with autism (Sharma et al., 2018). This high prevalence of co-occurring conditions adds complexity to the study and understanding of sources of the possible difficulties experienced by autistic individuals.

### Current study

While Sturrock et al. (2022) conducted qualitative interviews with a small sample of people on the autistic spectrum, they did not include a control group. Given the diversity of individual profiles within autism, and within the non-autistic population, it is important to determine whether auditory challenges are more prevalent in autistic individuals than non-autistic individuals, as this can help direct future research and resources toward challenges that may be less recognized. This study, therefore, aims to expand the findings of Sturrock et al. (2022) by systematically quantifying the prevalence and nature of auditory difficulties in daily life situations among individuals with autism compared with controls without autism. This was done by asking people in a self-report questionnaire about their experience perceiving speech, locating origins of sounds as well as the qualities of the sounds they hear with the 15-item Speech, Spatial, and Qualities of Hearing Questionnaire (15-SSQ). This study aims to contribute to a better understanding of the prevalence and impact of auditory challenges experienced by autistic adults by testing (1) whether autistic adults, compared with non-autistic adults, report different perceived hearing, both in general and specifically regarding speech, spatial processing, and quality. To provide a more inclusive research sampling, and to assess the impact of diagnostic status as an inclusion criterion for autism research, we also (2) separately assessed self-reporting hearing ability in individuals with a clinical diagnosis of autism and those who self-identify as autistic.

### Methods

#### Participant characteristics

In total, 137 individuals participated in the study. Of these, the present analyses exclude eight participants who reported having a hearing impairment, leaving 129 participants in the final analyses. The participants who reported a hearing impairment reported the following: two Tinnitus, two hearing impairments in one ear, and two auditory processing disorder. Among the 129 participants, 45 (32.8%) reported having a clinical diagnosis of autism. In addition, 18 respondents (13.1%) reported they self-identify as autistic, of which six were in the process of seeking a clinical diagnosis. The remaining 66 respondents (48.2%) confirmed they did not have autism. Due to the online nature of this study, and our motivation to maintain a low participation burden on the participants, we are unable to confirm the clinical diagnoses. Self-identification was based solely on participant report and was not verified using standardized autism screening tools or clinical assessments. This approach was chosen to maintain a low participation burden and facilitate broad international recruitment. We acknowledge that self-identification reflects participants’ own understanding of their status, which may be influenced by diverse personal and contextual factors. Table 1 provides an overview of participant characteristics per group.

**Table 1.** Participant characteristics.

	N	Mean age (*SD*)	Gender	Presence of co-occurring conditions
Autism (clinical diagnosis)	45	34.0 ± 11.3 years	23 female, 17 male, 5 non-binary or third gender	3 ADHD, 2 Depression, 2 Anxiety, 1 Social Pragmatic Communication Disorder
Autism (self- identifying)	18	33.0 ± 11.0 years	12 female, 2 male, 2 non-binary or third gender	5 ADHD, 1 Depression
No autism	66	30.8 ± 11.2 years	53 females, 8 male, 5 non-binary or third gender	6 ADHD, 1 Anxiety, 1 OCD

Participants were recruited through flyers and online advertisements and were invited to participate regardless of country of origin or residence, or native speaker status. The only exclusion criterion was the presence of a hearing impairment. This wide sampling procedure was chosen in order to have a more inclusive sample. Given that auditory processing is not expected to be culture- or language-dependent, we believe the research question also lends itself to a wider sampling procedure. As there was no monetary reward for participation and we aimed for a sample that is as representative as possible, there was no target sample size. The questionnaire was left open for as long as possible within the limits of the project timeline in which this study took place.

While our participant sampling was intentionally international in order to provide a greater degree of geographic diversity, we did not have a priori hypotheses regarding participant demographics. We, therefore, provide an overview of country of residence, native language, and country of birth in Supplementary Appendix A, corresponding to Supplementary Figures 1–3, respectively.

Participants gave their written consent to each procedure before beginning. The study was carried out in conformity with the Declaration of Helsinki and was approved by the Ethics Committee Social Sciences of Radboud University Nijmegen (ethical approval number: ECSW-2023-090).

The participants were not financially reimbursed for their participation.

### Procedure and questionnaires

All participants first filled in the 15-SSQ questionnaire and provided information about demographics, such as age, gender, diagnoses,^2^ country of birth, country of residence, native language, and hearing impairment. The inclusion of the question regarding hearing impairments was aimed to control for confounds in auditory processing that are not necessarily related to autism. The questionnaire was completed through Qualtrics.

### 15-SSQ questionnaire

The SSQ was designed to measure subjective hearing ability across a range of domains (Gatehouse & Noble, 2004). The 15-SSQ (Moulin et al., 2019), which was used in this study, is a condensed version of the test that consists of 15 items subdivided into three domains: five addressing speech hearing (e.g. “You are in a group of about five people in a busy restaurant. You can see everyone else in the group. Can you follow the conversation?”), five concerning spatial hearing (e.g. “You are standing on the footpath of a busy street. Can you hear right away which direction a bus or truck is coming from before you see it?”), and five to measure the quality level of the sounds heard (e.g. “Do everyday sounds that you can hear easily seem clear to you (not blurred)?”). Through a response slider, participants assessed how much they agreed with the statement on a range of 0 (not at all) to 10 (perfectly). In this questionnaire, a higher score indicates a better perceived hearing ability. The 15-SSQ has previously been shown to have good internal reliability (Cronbach’s α = 0.94 for full scale, 0.92 for Speech subscale, 0.96 for Spatial subscale, and 0.91 for Quality subscale) (Moulin et al., 2019). Construct validity has also been shown through significant correlation with hearing loss (β = 0.81) (Moulin et al., 2019). The complete questionnaire can be found in Supplementary Appendix B, in Supplementary Table 1.

The total score of the 15-SSQ was determined by averaging each participant’s responses to the 15 items. Similarly, by averaging each participant’s responses within the same subcategory, the subscore averages were calculated.

### Statistical analysis

Analyses were performed with the R statistical software (v.4.4.1; R Core Team, 2024). We assessed the association between group and SSQ (subscale) score using linear regression, modeling SSQ score as the dependent variable, subscale (including SSQ_full, Quality, Spatial, and Hearing) and group (no autism, autism clinical-dx, autism self-dx) as primary independent variables of interest, and presence of co-occurring condition (binary, yes/no) and age as additional independent variables. Co-occurring conditions were binarized as there would otherwise be insufficient statistical power to assess the impact of different conditions. Plots were generated using the R packages *ggplot2* (Wickham, 2016), *sjPlot* (Lüdecke, 2025), and *wesanderson* (Ram & Wickham, 2023). The *emmeans* package (Lenth & Piaskowski, 2025) was used to conduct Tukey-adjusted post hoc *t* tests to assess differences between the clinically diagnosed and self-identifying autism groups. Specifically, *emmeans* calculates the estimated marginal means in its pairwise tests, thus maintaining any covariates. The TOSTER package (Caldwell, 2022; Lakens, 2017) was used for equivalence testing (i.e. evidence in favor of the null hypothesis) between groups in order to statistically assess the similarity between the clinically diagnosed and self-diagnosed autistic groups.

### Data availability

Data and code used in this study are openly available via the Open Science Framework: https://osf.io/rtq52/

### Results

Our model, which showed adjusted *R*^2^ of 0.386, showed a main effect of subscale, *F*(3) = 51.446, *p* < 0.001; group, *F*(2) = 70.127, *p* < 0.001; and an interaction between subscale and group, *F*(6) = 6.193, *p* < 0.001. We observed no effect of age, *F*(1) = 1.819, *p* = 0.178, or co-occurring diagnoses, *F*(1) = 0.002, *p* = 0.967. As an additional check, we also re-fitted the model while excluding any participants who reported having a co-occurring condition. This model showed the same significant effects, similar magnitudes of effect, and a similar adjusted *R*^2^ as the original model. Results of the post hoc contrasts, per SSQ subscale, are given below. These results are all computed using Tukey’s HSD, and thus the *p* values are corrected for the number of comparisons.

#### SSQ full score

**Figure 1.**
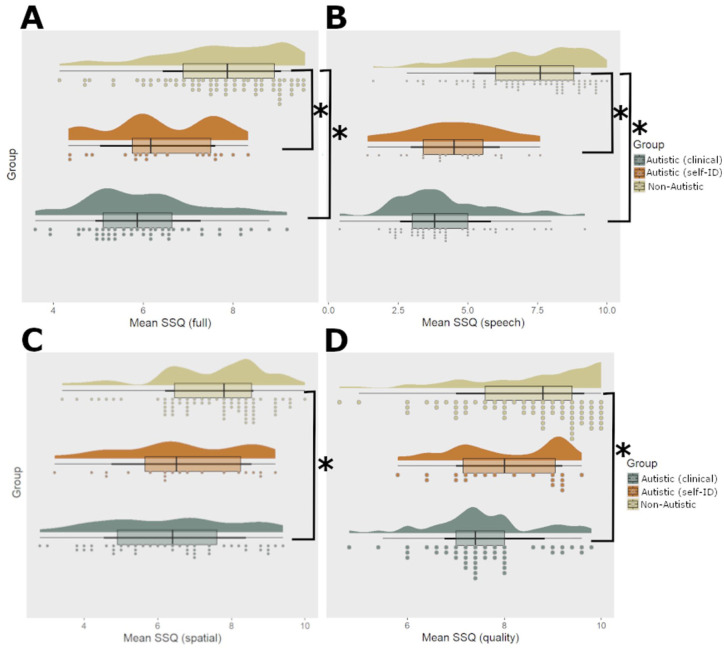
Overview of SSQ scores across participant groups. Panel **A** depicts the SSQ full score, panel **B** depicts the SSQ Speech subscale, panel **C** depicts the SSQ Spatial subscale, and panel **D** depicts the SSQ Quality subscale. In each panel, SSQ score is given on the x axis, while each group’s values are given as a separate “raincloud” on the y-axis. Rainclouds (Allen et al., 2021) provide the overall density distribution of the data via the filled “cloud” shape, the raw data points presented as circles underneath, and a boxplot in the center where the hinges (i.e. edges) correspond to the first and third quartiles, whiskers extend to the largest value that is no further than 1.5* the interquartile range from the hinge, and the center line provides the median of the data. * Indicates a significant difference (*p* < 0.05 after correction).

Compared with non-autistic participants, lower SSQ full scores were reported by those with a clinical diagnosis of autism (β = –1.708 (90% confidence interval (CI) = [1.09, 2.32]), *SE* = 0.298, *t* ratio = –5.726, *p* < 0.001) as well as those who self-identify as autistic (β = –1.380 (90% CI = [0.54, 2.22]), *SE* = 0.409, *t* ratio = –3.376, *p* = 0.002). No difference between clinically diagnosed and self-identifying autistic individuals was observed (*t* ratio = 0.765, *p* *=* 0.725), and follow-up analysis showed evidence for practical equivalence between the two groups (*t* = –3.461, *p* = 0.002) (see Figure 1(A)).

#### Speech hearing

Compared with non-autistic participants, lower SSQ speech scores were reported by those with a clinical diagnosis of autism (β = –3.001 (90% CI = [2.39, 3.62]), *SE* = 0.298, *t* ratio = –10.061, *p* < 0.001) as well as those who self- identify as autistic (β = –2.781 (90% CI = [1.94, 3.62]), *SE* = 0.409, *t* ratio = –6.803, *p* < 0.001). No difference between clinically diagnosed and self-identifying autistic individuals was observed (*t* ratio = 0.491, *p* *=* 0.876) and follow-up analysis showed evidence for practical equivalence between the two groups (*t* = –2.433, *p* = 0.024). See Figure 1(B).

#### Spatial hearing

Compared with non-autistic participants, lower SSQ spatial scores were reported by those with a clinical diagnosis of autism (β = –1.231 (90% CI = [–0.62, –1.85]), *SE* = 0.298, *t* ratio = –4.128, *p* < 0.001), but no difference was seen between non-autistic and those who self- identify as autistic (β = –0.927, *SE* = 0.409, *t* ratio = –2.268, *p* = 0.061). No difference between clinically diagnosed and self-identifying autistic individuals was observed (*t* ratio = 0.304, *SE* = 0.429, *p* = 0.758), and follow-up analysis showed evidence for practical equivalence between the two groups (*t* = –3.353, *p* = 0.003). See Figure 1(C).

#### Qualities of hearing

Compared with non-autistic participants, lower SSQ spatial scores were reported by those with a clinical diagnosis of autism (β = –0.892 (90% CI = [–0.28, –1.51]), *SE* = 0.298, *t* = –2.990, *p* = 0.008). No difference between non-autistic and self-identifying autistic individuals (*t* ratio = 1.057, *p* = 0.541), or between clinically diagnosed and self-identifying autistic individuals was observed (*t* ratio = 1.025, *p* = 0.562), and follow-up analysis showed evidence for practical equivalence between the clinically diagnosed and self-identifying individuals (*t* = –4.975, *p* < 0.001), as well as practical equivalence between the non-autistic and self-identifying autistic individuals (*t* = 4.840, *p* < 0.001). See Figure 1(D).

#### Post hoc analysis: differences between hearing type

As an additional, post hoc analysis, we assessed whether there were statistically robust differences between the subscales. This follows from the observation that the parameter estimates for SSQ speech are larger than for SSQ spatial or quality. If autistic individuals report more difficulties with speech-specific hearing than other types of auditory perception, this would suggest that rather than very general auditory processing challenges or challenges associated with more pragmatic aspects of language processing, there is a specific difficulty with following others’ speech. To assess differences between hearing types, we contrast the marginal means of the subscale scores, within each group, using the emmeans package (Lenth & Piaskowski, 2025).

**Figure 2.**
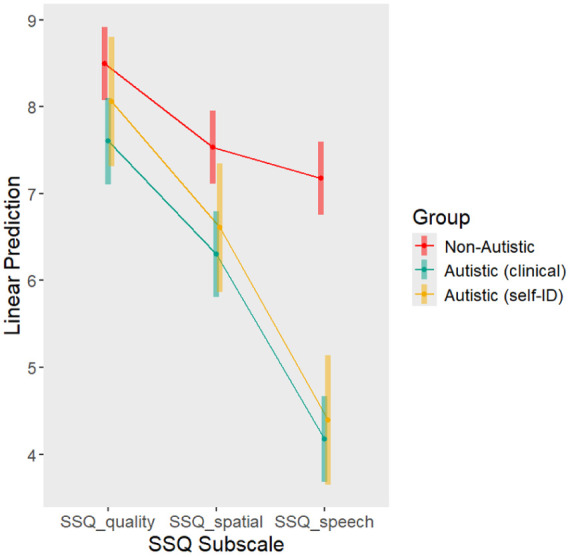
Marginal means of SSQ subscales. The three subscales are given on the x-axis, while the predicted (marginal) SSQ subscale scores are given on the y-axis. Groups are indicated as separate lines. As described in Table 2, all between-subscale differences were significant, with the exception of *spatial—speech* in the non-autistic group.

We found that across all groups, SSQ quality was rated higher than SSQ spatial or speech. However, both self-identifying and clinically diagnosed autistic participants also rated SSQ speech as lower than SSQ spatial. See Table 2 and Figure 2 for an overview of these results.

**Table 2.** Overview of subscale contrast analysis.

Contrast	Estimate [lower, upper-confidence limit]	*SE*	t-ratio	p-value
No-autism				
Quality—Spatial	0.961 [0.28, 1.65]	0.278	3.456	0.002^a^
Quality—Speech	1.318 [0.71, 1.93]	0.278	4.742	< 0.001^a^
Spatial—Speech	0.358 [-0.25, 0.97]	0.278	1.286	0.404
Autism (clinical dx)				
Quality—Spatial	1.300 [0.55, 2.05]	0.340	3.819	< 0.001^a^
Quality—Speech	3.427 [2.68, 4.18]	0.340	10.067	< 0.001^a^
Spatial—Speech	2.127 [1.38, 2.88]	0.340	6.249	< 0.001^a^
Autism (self-dx)				
Quality—Spatial	1.456 [0.28, 2.63]	0.532	2.735	0.018^a^
Quality—Speech	3.667 [2.49, 4.84]	0.532	6.889	< 0.001^a^
Spatial—Speech	2.211 [1.04, 3.38]	0.532	4.154	< 0.001^a^

a Indicates statistical significance. Confidence limits use 90% confidence interval.

## Discussion

This study assessed how auditory difficulties in daily life differ between autistic and non-autistic individuals. Given the prevalence of self-identification in the autism community, we also assessed whether there were differences between self-identifying and clinically diagnosed autistic adults. Both clinically diagnosed and self-identifying autistic individuals reported significantly lower scores in the Speech subscale and the full scale, compared with non-autistic participants. In the Quality of Hearing and Spatial Hearing subscales, clinically diagnosed autistic individuals reported significantly lower scores than the non-autistic group while self-identifying adults did not show differences from the group without autism. Notably, both the clinically diagnosed and self- identifying groups reported comparable experiences in auditory difficulties. Finally, post hoc analysis shows that autistic individuals have most difficulties with speech hearing.

### Differences in hearing

The findings consistently demonstrate that both clinically diagnosed and self-identifying individuals with autism exhibit significantly lower scores compared with non-autistic individuals on measures of auditory processing. Specifically, across clinically- and self-identifying individuals, autistic participants reported lower SSQ_full and Speech Hearing scores, indicating challenges in overall auditory perception. This aligns with the recent interview data suggesting that autistic individuals experience difficulties in processing auditory information comprehensively (Sturrock et al., 2022). Our findings are also in line with recent work by Bang and Igelström (2024), who found similar scores on the SSQ in autistic adults. While Bang and Igelström used the full SSQ and a different division of subscales (i.e. Speech Understanding, Spatial Perception, Clarity and Separation, and Listening Effort), the scores for their first three subscales (4.59, 5.47, and 7.20) are quite similar to the scores we found for the Speech, Spatial, and Qualities subscales of the 15-SSQ.

Previous research suggests that sensory dysregulation, particularly in the auditory domain, can contribute to social difficulties in autistic individuals. Specifically, sensory sensitivities can impair the ability to decode social cues, such as prosody and intonation, making it difficult to understand the intentions and emotions of others (Thye et al., 2018). The challenges in processing these auditory cues may, in turn, hinder social reciprocity and contribute to the systematic avoidance of social interactions and certain environments, further exacerbating social difficulties (Leekam et al., 2006; Robertson & Simmons, 2012; Tavassoli et al., 2014). Beyond understanding emotions and intentions from auditory cues, our results also show that many autistic individuals experience difficulties in auditory perception more generally. Critically, these differences are not just “statistically robust but clinically insignificant” differences. A difference of at least 1.0 on any of the subscales is considered a clinically relevant difference (Cowan et al., 2024), and our results predict a 1+ difference between autistic and non-autistic individuals in the spatial subscale, and a difference of 2 to 3 points in the hearing scale. The observed lower SSQ scores in autistic participants, therefore, indicate a clinically meaningful difference in hearing ability, particularly related to speech. It is, therefore, important to note that these differences are seen in individuals without (reported) hearing impairments, which implies that current audiological testing may not fully capture the everyday hearing challenges faced by many autistic people. The observed SSQ score differences suggest that autistic individuals may face significant challenges in real-world auditory contexts, such as following conversations at noisy dinner tables or identifying sound sources in traffic. These findings may, therefore, have broad implications for the social functioning of autistic individuals who experience such difficulties. This supports the need for clinicians and educators to address such real-world auditory issues through targeted support strategies.

One promising avenue may be the use of hearing assistive technologies (HAT). Research by Xu et al. (2023) demonstrated that HAT use significantly improved sentence-in-noise recognition and reduced listening effort in autistic children, suggesting that such tools could help autistic individuals navigate noisy environments more comfortably and safely. While our study did not directly assess HAT use, the presence of speech hearing difficulties among participants supports the potential relevance of such interventions. It would be valuable for future research to investigate whether SSQ scores vary as a function of HAT use, particularly in adolescent and adult populations. Older individuals may face additional barriers related to workplace norms, stigma, or practical feasibility that influence the adoption and effectiveness of HAT in real-life settings. Studying these factors could help to contextualize the current findings and support the generalizability of results across age groups.

These findings suggest that speech hearing difficulties in autism may warrant further clinical attention and could represent an under-recognized aspect of the autistic experience. Increasing awareness of these challenges may also reduce self-doubt among autistic individuals who might experience real-life hearing issues despite normal audiogram results, and may contribute to the development of more holistic diagnostic and support frameworks.

Finally, our post hoc analysis revealed that contrary to the pattern seen in non-autistic individuals, participants with autism showed significantly lower scores for speech hearing than for spatial or quality of hearing. This suggests that for autistic individuals, speech understanding, aside from difficulties with pragmatics or affect recognition, may be generally more challenging at the level of auditory perception. This difficulty is also more pronounced than any broader auditory processing differences. This is a valuable finding because this means that for many autistic individuals, even if social situations are not prohibitively overstimulating, there is an additional challenge in keeping up with what is being said. This creates an extra barrier for social engagement. More research is, therefore, needed to better understand these speech hearing difficulties, and what can be done to reduce these barriers. In addition, it must be noted that these findings are based on self-report data, with no auditory testing being performed. Future research can also benefit from comparing self-reported hearing challenges with audiological data in order to better determine the source of experienced hearing challenges.

### Cross-linguistic considerations in SSQ responses

The SSQ was administered in English, although the linguistic background of the sample was highly diverse: 46% of participants reported Dutch as their native language, and the remainder represented a wide range of other languages. The complete geographic information of the sample can be found in Supplementary Appendix A.

While this might raise questions about cross-linguistic comparability, we consider the influence of native language phonological structures to be limited in this context. The SSQ items do not require understanding of specific words or phonemes, but rather focus on general auditory scenes, for example, being able to follow a conversation in background noise, localize a sound source, or distinguish between environmental sounds like buses or footsteps. These are situations encountered across cultures and do not rely on language-specific features. The consistent pattern of greater reported difficulties in the autistic group across this multilingual sample suggests that the findings are not tied to native phonology. Instead, they may reflect broader auditory perceptual differences that could be relevant across linguistic and cultural boundaries. However, future cross-linguistic comparisons may be warranted for languages that are known to differ in terms of the informativity of the acoustic signal compared to contextual context (e.g. as in the case of Danish (Ishkhanyan et al., 2019; Trecca et al., 2019)).

### Similarity between clinical-diagnosed and self-identifying

For the full SSQ score as well as the SSQ_speech subscale, both clinically diagnosed and self- identifying individuals with autism reported lower scores compared with their non-autistic counterparts. While clinically diagnosed individuals showed significant differences in the SSQ_qualities and SSQ_spatial subscales when compared with the non-autistic participants—indicating lower scores related to perceived sound quality—such differences were not observed among self-identifying individuals. For both of these subscales, there was also no difference observed between the clinically diagnosed and self-identifying groups. In fact, equivalence testing showed statistical evidence for the two groups to be practically equivalent using the same 1.0 point difference as the minimum for clinically relevant difference as described earlier (Cowan et al., 2024). This suggests a nuanced auditory experience within the autistic population, where self- identifying individuals may perceive sound quality more similarly to non-autistic individuals than clinically diagnosed individuals. However, the lack of differences observed between the two autism groups further indicates that the self-identifying group may be somewhere in between the clinically diagnosed and non-autistic groups in terms of their hearing experience. It should additionally be noted that the self-identifying group was substantially smaller than the clinical diagnosis group, which may have led to additional variance, and thus limiting the statistical power and generalizability of these comparisons. The inclusion of self-identifying autistic participants, while increasing the inclusivity and relevance of our study, introduced variability due to the absence of formal clinical confirmation. Self-identification in this context reflected individuals’ own recognition of autistic traits or experiences, which can vary widely depending on personal insight, access to diagnostic resources, and cultural factors. Future studies with larger samples and the inclusion of standardized screening tools would help clarify these relationships further and improve generalizability. Within the limits of our study, we can at least conclude that both self-identifying and clinically diagnosed autistic individuals experience more challenges associated with auditory perception, particularly in the domain of speech hearing. While there are no studies that have explicitly compared clinically diagnosed and self-identifying individuals in terms of auditory perception, our results are in line with the broader finding of similarity between self-identifying and clinically diagnosed autistic adults, at least in terms of autism screener questionnaires (Sturm et al., 2024) and self-reported experiences of stigma and quality of life (McDonald, 2020). Future studies with larger and more balanced samples of self-identifying and clinically diagnosed individuals could better elucidate whether the observed similarities reflect true overlap or are partially due to sample size limitations. Further exploration into the factors influencing self-identification and its implications on auditory experiences is recommended to better understand these dynamics.

### Personal experiences and future directions

This survey provides an initial indication of the difficulties faced by autistic individuals in terms of auditory processing. Future research should follow up on these findings to better understand the mechanism behind these auditory processing difficulties. For example, one participant noted that background noise does not necessarily make it more difficult to *hear* their partner, but rather the background noise is processed “with the same priority” as the speech of their partner. In other words, the speech is not masked by the background noise, but rather there seems to be a difficulty in selectively attending to one person’s speech. Similarly, another participant suggested that while it is possible to find where and who is talking while being in a group and in crowded areas, it is nevertheless difficult to quickly understand what the other person is saying. If the other person is talking at a fast pace, the participant, diagnosed with Autism, mentioned that it will take a longer time to “decipher” what the other person is saying because of the high volume of stimuli that need to be processed at the same time. Indicating that, in their personal experience, the amount of stimuli affects the processing speed of information. Beyond that, energy costs (i.e. the mental and physical effort required to engage in and maintain a conversation), tiredness, and feeling overwhelmed were reported by the participants to influence their speech processing together with the amount of stimuli and length of the conversation. This was partly supported by the findings of Kwakye et al. (2011), where participants heard two identical clicks (one per ear) in close temporal proximity, and were then asked which ear the first click was presented to. In their study, children with autism needed 48% longer time between auditory stimuli to accurately identify which click happened first, compared with non-autistic peers. These findings can provide a foundation for future research and the development of support strategies tailored to the sensory needs of autistic individuals.

### Contribution of the present study

Overall, our findings indicate that auditory perception challenges in autism are not limited to specific speech cues but extend more broadly to everyday situations, especially speech understanding. Using the short-form SSQ-15, we show that these differences can be captured effectively with a concise tool, supporting its value for future research across diverse populations, as demonstrated here in an international sample recruited in English from the Netherlands and beyond. We also found similar patterns in both self-identifying and clinically diagnosed autistic adults, highlighting the shared experiences across the autistic community. These results, rooted in the everyday listening situations described by autistic adults, underscore the practical importance of recognizing auditory perception as a meaningful aspect of many autistic people’s communication and well-being.

## Conclusion

In conclusion, this study investigated auditory perception in autism using the 15-SSQ questionnaire across various domains. Overall, this study showed that autistic individuals, whether self-identifying or clinically diagnosed, reported experiencing significantly reduced hearing quality in daily life situations, particularly related to understanding speech. These results underscore the pervasive challenges faced by individuals on the autism spectrum in processing auditory information in general and speech in particular. These findings highlight a need for more research, and more awareness regarding accommodations, for general auditory perception challenges faced by many autistic individuals.

## Positionality statement

**ESS** is a neurodivergent cognitive neuroscientist and clinical neuropsychologist. She grounds her work in autistic people’s lived experiences, which guide research questions and methods. The autism community experiences shape both the focus and approach of her work. **LD** is a cognitive neuroscientist and psycholinguist who engaged with autistic researchers and the autistic community to shape her previous and current work involving this group. **JPT** is an autistic communication researcher who studies communication through the lens of neurodiversity, and engages with autistic researchers, advisory groups, and the broader autism community to better situate his research.

## Supplemental Material

sj-docx-1-aut-10.1177_13623613251391492 – Supplemental material for Exploring auditory perception experiences in daily situations in autistic adults Supplemental material, sj-docx-1-aut-10.1177_13623613251391492 for Exploring auditory perception experiences in daily situations in autistic adults by Elena Sofia Silva, Linda Drijvers and James P Trujillo in Autism

## Notes

1. There has been a growing debate over the terminology used to describe autism, particularly between person-first (PFL) (e.g. “person with autism”) and identity-first (IFL) (e.g. “autistic person”) language (Keating et al., 2022). Keating et al. (2022) expanded this debate by examining various autism-related terms and the language preferences of English-speaking autistic individuals globally. Their mixed-methods study found consistent preferences for terms like “Autism,” “Autistic person,” and “Neurological/Brain Difference.” In addition, the authors emphasized respect for individual preferences and the absence of a universally correct way to talk about autism. However, preferences for PFL have been observed in Australia (Bury et al., 2020) and the Netherlands (Buijsman et al., 2022). In addition, the study conducted in the Netherlands by Bosman and Thijs (2024) revealed that language preferences still vary greatly among individuals in the Dutch autism community. We, therefore, use IFL and PFL interchangeably, respecting the variances of preferences worldwide.

2. Regarding autism diagnosis, participants were asked, “Do you have an Autism Spectrum Disorder (ASD) diagnosis? (tick what is applicable to you)” with the following response options: “Yes, clinically diagnosed,” “Yes, self-diagnosed,” “Not yet, I am in the process to get an ASD diagnosis,” “I have another diagnosis: __,” and “No.” The option “Yes, self-diagnosed” was included to allow individuals who identify as autistic without a formal clinical diagnosis to affirm their status, thereby promoting inclusivity. For clarity and respect, participants who chose this option are referred to as “self-identifying” in this study, emphasizing self-knowledge and lived experience rather than clinical validation.
