# Supplementary material for: Exploring auditory perception experiences in daily situations in autistic adults
Source: Autism. 2025 Nov 14;30(2):439–51. doi: 10.1177/13623613251391492 (PMC12804400; doi:10.1177/13623613251391492)
Supplement: sj-docx-1-aut-10.1177_13623613251391492 – Supplemental material for Exploring auditory perception experiences in daily situations in autistic adults [file sj-docx-1-aut-10.1177_13623613251391492.docx]

**Appendix A**

**Geographic information of the sample**

**Supplemental Figure 1.** *Country of residence*


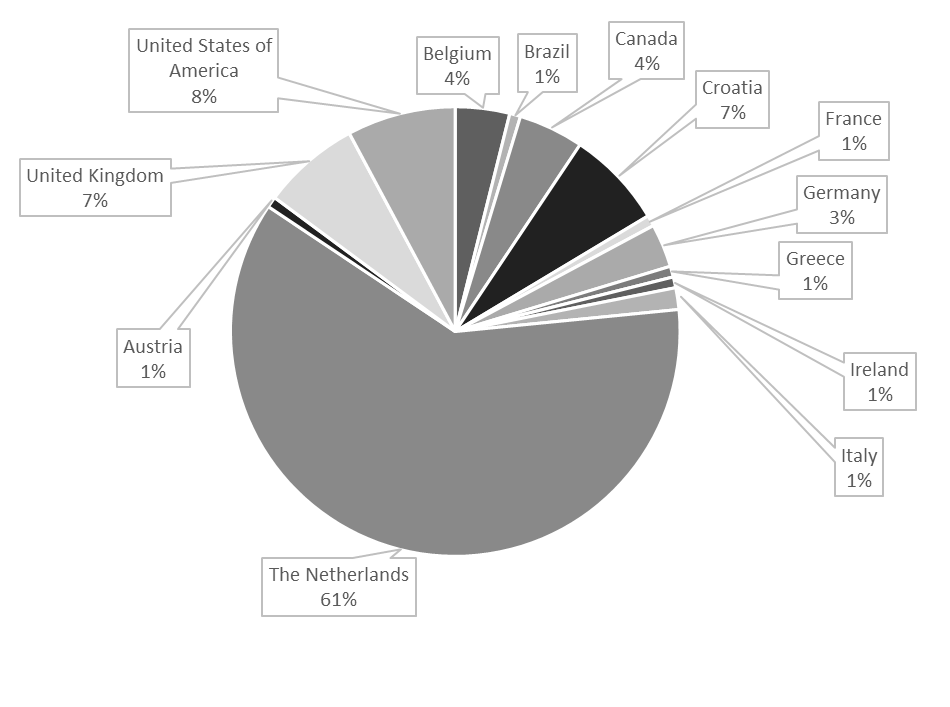


## Supplemental Figure 2. *Participant native languages*

*
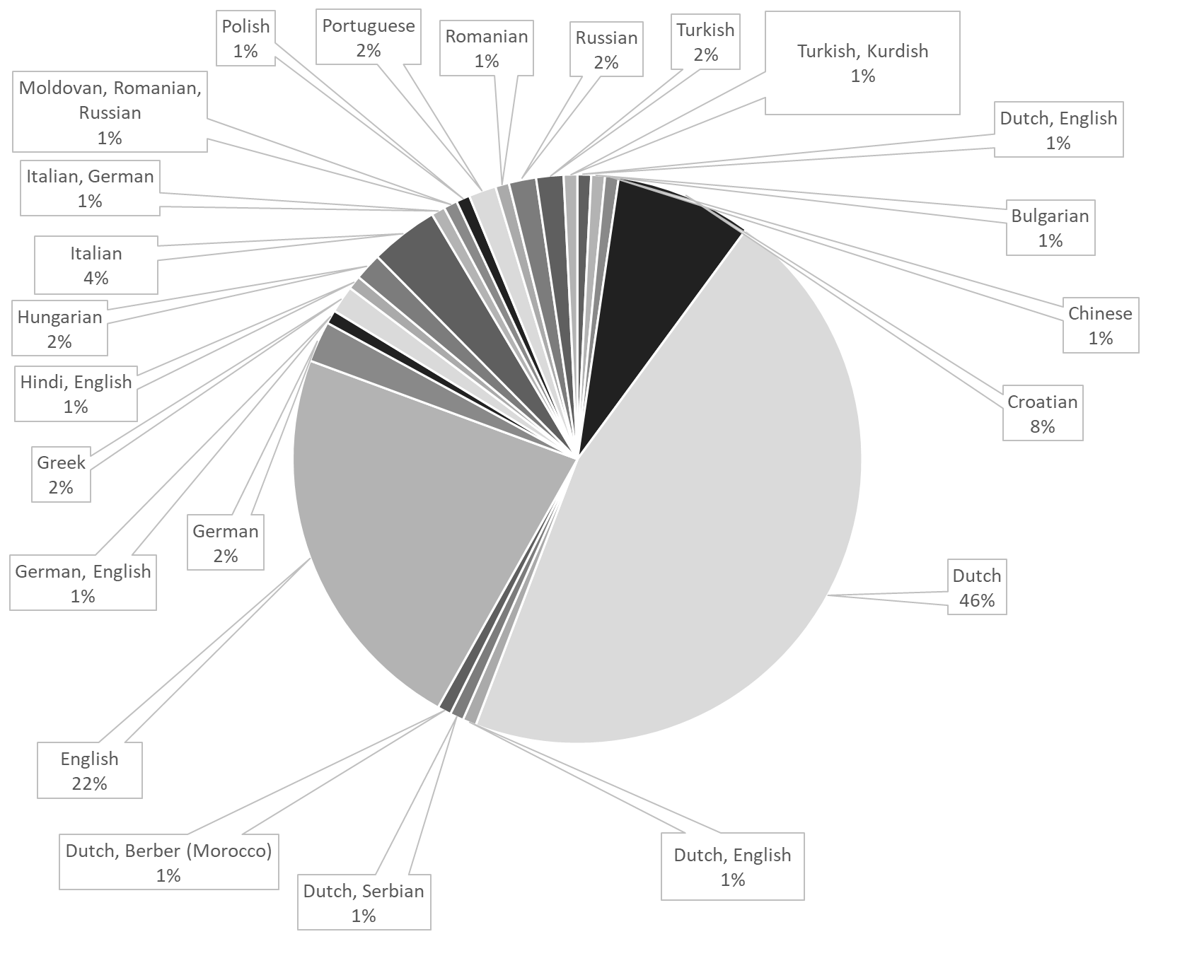
*

## Supplemental Figure 3. *Country of birth*


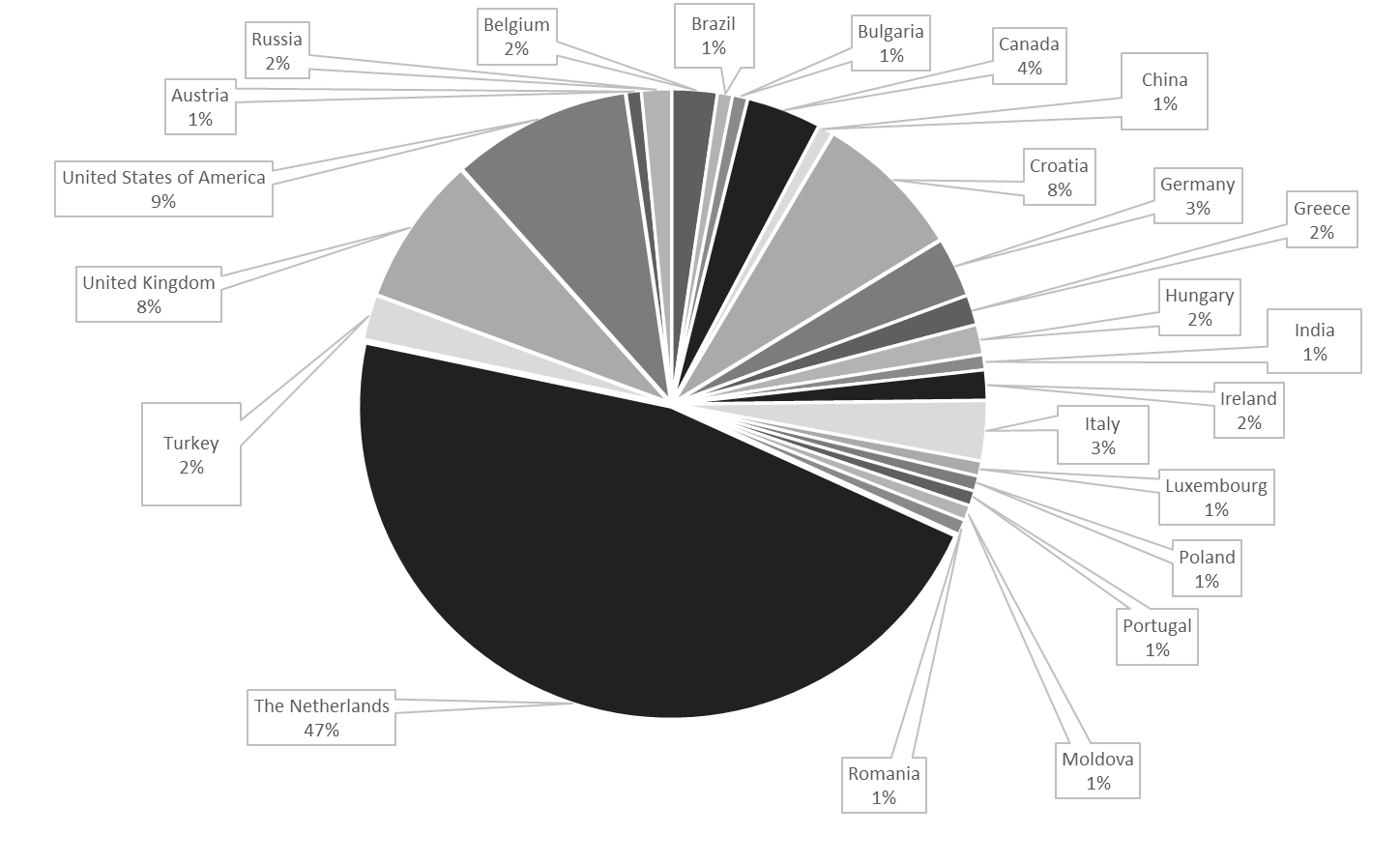


## Appendix B 15 items SSQ

**Supplementary Table 1**

*Complete 15-SSQ questionnaire items*

| **15-SSQ**  **Item** | **Vignette** | **Anchors** |
| --- | --- | --- |
| Speech 1 | You are talking with one other person and there is a TV on in the same room.  Without turning the TV down, can you follow what the person you’re talking to says? | Not at all–Perfectly |
| Speech 2 | You are in a group of about five people in a busy restaurant. You can see everyone else in the group.  Can you follow the conversation? | Not at all–Perfectly |
| Speech 3 | You are talking with one other person. There is continuous background noise, such as a fan or running water.  Can you follow what the person says? | Not at all–Perfectly |
| Speech 4 | You are in a group of about five people in a busy restaurant. You cannot see everyone else in the group.  Can you follow the conversation? | Not at all–Perfectly |
| Speech 5 | You are in conversation with one person in a room where there are many other people talking.  Can you follow what the person you are talking to is saying? | Not at all–Perfectly |
| Spatial 1 | You are sitting around a table or at a meeting with several people. You can’t see everyone.  Can you tell where any person is as soon as they start speaking? | Not at all–Perfectly |
| Spatial 2 | You are outside. A dog barks loudly.  Can you tell immediately where it is, without having to look? | Not at all–Perfectly |
| Spatial 3 | You are standing on the footpath of a busy street.  Can you hear right away which direction a bus or truck is coming from before you see it? | Not at all–Perfectly |
| Spatial 4 | Can you tell from the sound of their voice or footsteps which direction a person is moving, e.g. from your left to your right or right to left? | Not at all–Perfectly |
| Spatial 5 | Do you have the impression of sounds being exactly where you would expect them to be? | Not at all–Perfectly |
| Qualities 1 | Do you find it easy to recognize different people you know by the sound of each one’s voice? | Not at all–Perfectly |
| Qualities 2 | Do you find it easy to distinguish different pieces of music that you are familiar with? | Not at all–Perfectly |
| Qualities 3 | Can you tell the difference between different sounds, e.g. a car versus a bus, or water boiling in a pot versus food cooking in a frying plan? | Not at all–Perfectly |
| Qualities 4 | When you listen to music, does it sound clear and natural? | Not at all–Perfectly |
| Qualities 5 | Do everyday sounds that you can hear easily seem clear to you (not blurred)? | Not at all–Perfectly |

*Note.* The items above are listed grouped by subscale but they were presented to the respondents in random order.
